# β-Amyloid promotes accumulation of lipid peroxides by inhibiting CD36-mediated clearance of oxidized lipoproteins

**DOI:** 10.1186/1742-2094-1-23

**Published:** 2004-11-16

**Authors:** Vidya V Kunjathoor, Anita A Tseng, Lea A Medeiros, Tayeba Khan, Kathryn J Moore

**Affiliations:** 1 Lipid Metabolism Unit, Dept. of Medicine, Massachusetts General Hospital, Harvard Medical School, Boston, MA, 02114 USA

## Abstract

**Background:**

Recent studies suggest that hypercholesterolemia, an established risk factor for atherosclerosis, is also a risk factor for Alzheimer's disease. The myeloid scavenger receptor CD36 binds oxidized lipoproteins that accumulate with hypercholesterolemia and mediates their clearance from the circulation and peripheral tissues. Recently, we demonstrated that CD36 also binds fibrillar β-amyloid and initiates a signaling cascade that regulates microglial recruitment and activation. As increased lipoprotein oxidation and accumulation of lipid peroxidation products have been reported in Alzheimer's disease, we investigated whether β-amyloid altered oxidized lipoprotein clearance via CD36.

**Methods:**

The availability of mice genetically deficient in class A (SRAI & II) and class B (CD36) scavenger receptors has facilitated studies to discriminate their individual actions. Using primary microglia and macrophages, we assessed the impact of Aβ on: (a) cholesterol ester accumulation by GC-MS and neutral lipid staining, (b) binding, uptake and degradation of ^125^I-labeled oxidized lipoproteins via CD36, SR-A and CD36/SR-A-independent pathways, (c) expression of SR-A and CD36. In addition, using mice with targeted deletions in essential kinases in the CD36-signaling cascade, we investigated whether Aβ-CD36 signaling altered metabolism of oxidized lipoproteins.

**Results:**

In primary microglia and macrophages, Aβ inhibited binding, uptake and degradation of oxidized low density lipoprotein (oxLDL) in a dose-dependent manner. While untreated cells accumulated abundant cholesterol ester in the presence of oxLDL, cells treated with Aβ were devoid of cholesterol ester. Pretreatment of cells with Aβ did not affect subsequent degradation of oxidized lipoproteins, indicating that lysosomal accumulation of Aβ did not disrupt this degradation pathway. Using mice with targeted deletions of the scavenger receptors, we demonstrated that Aβ inhibited oxidized lipoprotein binding and its subsequent degradation via CD36, but not SRA, and this was independent of Aβ-CD36-signaling. Furthermore, Aβ treatment decreased CD36, but not SRA, mRNA and protein, thereby reducing cell surface expression of this oxLDL receptor.

**Conclusions:**

Together, these data demonstrate that in the presence of β-amyloid, CD36-mediated clearance of oxidized lipoproteins is abrogated, which would promote the extracellular accumulation of these pro-inflammatory lipids and perpetuate lipid peroxidation.

## Background

Hypercholesterolemia is an established risk factor for atherosclerosis and a number of recent epidemiological studies have suggested a link between increased circulating cholesterol levels and Alzheimer's disease (AD) [1]. Lipoproteins in the serum and the central nervous system (CNS) mediate cholesterol homeostasis through the delivery and removal of cellular cholesterol. With hypercholesterolemia, these phospholipid and cholesterol rich-particles accumulate abnormally outside the arterial lumen, where they are susceptible to oxidization [2]. Lipoprotein-derived oxidation products (hydroperoxides, lysophosphatidylcholine, oxysterols and aldehydes) initiate the inflammatory response that drives atherosclerotic plaque formation in the artery wall, and these lipid peroxidation products, including malondialdehyde and 4-hydroxynonal (HNE), have also been detected in AD-affected brains [3,4]. AD patients have been reported to have cholesterol profiles known to be pro-atherosclerotic, including increased total serum and low-density lipoprotein (LDL) cholesterol, and increased susceptibility to lipoprotein oxidation [5-9]. Antibodies raised against oxidized LDL (oxLDL) demonstrate reactivity to amyloid plaques and surrounding tissue, indicating that lipid peroxidation epitopes present in oxLDL accumulate in the brains of AD patients [3]. Recently, oxidized cholesterol metabolites identified in both atherosclerotic and senile plaques have been found to accelerate β-amyloid fibril formation [10]. Together, these findings suggest that, as in atherosclerosis, the accumulation of lipoprotein oxidation products in Alzheimer's disease may contribute to chronic inflammation.

Phagocyte expressed pattern recognition receptors (PRR) are the first line of defense of the innate immune system against foreign or modified proteins and lipids. Scavenger receptors are pattern recognition receptors that bind and internalize a wide range of ligands, including certain polyanions, modified forms of LDL, advanced glycation endproducts and apoptotic cells [11]. These receptors are expressed by macrophages and microglia, and are the primary clearance pathway for pro-inflammatory oxidized lipoproteins [12]. In addition to binding oxLDL, several members of the scavenger receptor A (SRA) and B (CD36, SR-B1) class recognize fibrillar β-amyloid (Aβ), which accumulates in the brain and cerebral blood vessels in AD, as well as in coronary atherosclerotic plaques [13-15]. While studies in *Sra *null mice have failed to show a role for this receptor in the pathogenesis of AD [16], it has recently been demonstrated in our lab, and others, that Aβ activates an inflammatory signaling cascade via CD36 that regulates microglial activation and recruitment in the brain [17-19]. In AD patients, increased CD36 expression was detected in the frontal cortex which correlated with the presence of amyloid plaques and oxidative markers, suggesting that upregulation of this scavenger receptor pathway may also promote inflammation *in vivo *[20]. Similar to its role in peripheral macrophages, CD36 on microglia is believed to scavenge modified proteins and oxidized phospholipids. We hypothesized that a simultaneous increase in lipoprotein oxidation and accumulation of Aβ in the brain and blood vessels in AD might compromise the ability of this scavenger receptor to effectively clear these modified host ligands.

Aβ has previously been shown to reduce uptake of LDL modified by acetylation, in microglia and SRA- or SR-B1-transfected cells [21]. We have shown that CD36 binds acetylated LDL with very low affinity, indicating that these studies primarily addressed the impact of Aβ on Class A scavenger receptor activity [12]. Unlike SR-A, which binds the modified apolipoprotein B component of acetylated LDL, CD36 recognizes oxidized phospholipids within the oxidized lipoprotein particle [22]. CNS lipoproteins isolated from cerebrospinal fluid, astrocytes or microglia, contain similar amounts of phospholipid, cholesterol, and cholesteryl ester content as their serum counterparts, and a pro-oxidative environment in Alzheimer's disease is believed to accelerate the formation of lipid peroxides in these particles [23]. In this study, we assessed the impact of Aβ on the binding and degradation of oxLDL via CD36, SR-A and CD36/SR-A-independent pathways. The availability of mice genetically deficient in *Sra *and *Cd36 *has facilitated studies to discriminate the actions of these individual scavenger receptors. We show that Aβ dose-dependently inhibits oxLDL binding, lysosomal degradation and cholesterol ester accumulation in macrophages and microglia. This inhibitory effect was mediated specifically via CD36 and could be reversed by removal of extracellular Aβ, indicating that the lysosomal degradation pathway was not directly impaired. Furthermore, activation of CD36-signaling by Aβ did not mediate this inhibitory effect, as targeted inactivation of essential downstream kinases did not restore oxLDL degradation. Together, these data demonstrate that Aβ impairs the ability of CD36 to scavenge oxidized lipids by competing for receptor binding. This suggests that accumulation of Aβ in the brain and vessel wall in AD would inhibit the clearance of pro-inflammatory oxidized phospholipids and oxidized-phospholipid-containing particles such as lipoproteins, thereby promoting lipid peroxidation.

## Methods

### β-Amyloid

Aβ_1-42 _and reverse Aβ_42-1 _(*rev*Aβ) peptides were obtained from Biosource International (Camarillo, California). To induce fibril formation, Aβ_1-42 _was resuspended in H_2_O at 1 mg/ml and incubated for 1 week (37°C) and fibril formation was confirmed by thioflavine S (Sigma-Aldrich Co., St. Louis, Missouri) fluorescent staining as we previously described [17,18].

### Mice

The *Cd36*^-/- ^mice were generated in our laboratory as previously described [17] and SraI/II null (*Sra*^-/-^) mice were generously provided from Dr. T. Kodama (University of Tokyo, Japan) [24]. Both mouse lines were backcrossed to C57BL/6 mice for 7 generations (98.6% C57BL/6) prior to intercrossing to generate mice lacking both *Sra *and *Cd36*. Double knockout mice (*Sra*^-/-^/*Cd36*^-/-^) were generated from heterozygote intercrosses at the expected ration of 1:16. Wild type age-matched C57BL/6 mice (The Jackson Laboratory, Bar Harbor, Maine) were used as controls for these three lines. Lyn^-/- ^and Fyn^-/- ^mice were obtained from The Jackson Laboratory and Lyn^-/-^, Fyn^-/- ^and wild type littermate control mice were generated from heterozygote intercrosses. All mice were maintained in a pathogen-free facility with free access to rodent chow and water. All experimental procedures were carried out in accordance with Massachusetts General Hospital's institutional guidelines for use of laboratory animals.

### Primary macrophage and microglial culture

Macrophages were collected from 6–8 week old mice by peritoneal lavage 4 days after *i.p. *injection with 3% thioglycollate as we previously described [17,25]. Cells were washed in PBS, cultured for 2 h in DMEM with 5% FCS, and washed again to remove non-adherent cells. Adherent cells were incubated in DMEM with 1% FCS overnight prior to use and were routinely >95% CD11b^+ ^and F4/80^+ ^as determined by flow cytometric analysis. Primary microglia were prepared from mixed brain cultures of neonatal mice as we previously described [17]. Briefly, whole brains were incubated in 0.25% trypsin and 1 mM EDTA (10 min, 25°C) and dissociated to obtain a single cell-suspension. Cells were washed in HBSS (4x, 10 min) and cultured in DMEM containing 10% FCS, 1% Fungizone for 10–12 days. Microglia accumulating above astrocyte monolayers were collected after gentle agitation, washed and incubated in DMEM with 1% FCS overnight prior to use. Microglia prepared in this manner were routinely >95% CR3^+ ^and express SR-A and CD36 [14,17,18].

### Lipoproteins

Human ^125^I-LDL and LDL (d = 1.019 - 1.063) were purchased from Biomedical Technologies (Stoughton, Massachusetts) and oxidized as we previously described [12,26]. LDL was diluted to 250 μg/ml, dialyzed against PBS at 4°C to remove EDTA, and then dialyzed against 5 μM CuSO_4 _in PBS at 37°C for 6 or 10 h. Oxidation was terminated by the addition of 50 μM butylated hydroxytoluene and 200 μM EDTA and oxLDL was used within 2 days of preparation. Moderately oxidized LDL (6 h oxidation) had a relative electrophoretic mobility of approximately 2.5–3 times that of native, unmodified LDL, whereas extensively oxidized LDL (10 h oxidation) had a relative mobility four times that of native LDL.

### ^125^I-OxLDL degradation, binding and uptake assays

Measurement of ^125^I-oxLDL binding, degradation and uptake was performed on confluent monolayers of peritoneal macrophages (7 × 10^5^) and microglia (5 × 10^5^) in 24 well plates as we previously described [12,26]. Briefly, 10 μg/ml of ^125^I-oxLDL was added to cells in the presence or absence of 30-fold excess unlabeled oxLDL, native LDL, Aβ_1-42_, or *rev*Aβ peptide for 5 h at 37°C. To measure ^125^I-oxLDL degradation, media were removed and assayed for TCA-soluble non-iodide degradation products. To measure ^125^I-oxLDL binding in the presence Aβ_1-42 _or *rev*Aβ, cells were washed 3x with 50 mM Tris pH 7.4, 0.15 N NaCl and 2 mg/ml BSA, 1x with 50 mM Tris pH 7.4 and 0.15 N NaCl and treated with 0.4% dextran sulfate to release surface bound ^125^I-oxLDL [27]. To measure ^125^I-oxLDL uptake, cells were washed 3x in 50 mM Tris pH 7.4 and 0.15 N NaCl, lysed in 0.1 N NaOH and assayed for ^125^I and cellular protein content. In some experiments, cell-association of oxLDL (cell-surface bound and endocytosed oxLDL) was measured by omitting the dextran sulfate treatment. Cellular protein content was measured by BCA assay (Pierce, Rockford, IL) and degradation, binding and uptake activity are expressed as ng ^125^I-oxLDL/mg protein. Specific degradation was calculated as the difference of total cellular degradation of ^125^I-oxLDL in the presence and absence of 30-fold excess unlabelled oxLDL competitor. All measurements were performed in triplicate and are representative of at least 3 experiments.

### Analysis of cellular cholesterol content

Macrophages and microglia were cultured with 40 μg/ml of oxLDL for 48 h in the presence or absence of Aβ_1-42 _or revAβ. Cholesterol ester accumulation was assessed by gas chromatography-mass spectrometry (GC-MS) and oil red O staining as we previously described [12,26]. For GC-MS analysis, lipids were extracted with hexane:isopropanol (3:2) and stigmasterol (Sigma, St. Louis, Missouri) was added as an internal standard. Lipid extracts were washed once with water and divided equally. One lipid aliquot was saponified for determination of total cholesterol and the second aliquot analyzed for free cholesterol using gas chromatography-mass spectrometry. The samples were injected (splitless) into an Agilent 6890 GC-MS-(G2613A system, Agilent Technologies, Palo Alto, CA) equipped with a J&W DB17 fused silica capillary column (15 m × 0.25 mm inner diameter × 0.5 μm; J&W Scientific, Folsom, CA). The GC temperature program was as follows: the initial temperature was 260°C for 5 min, then increased to 280°C (5°C/min) and held 280°C for 11 min. A model 5973N mass-selective detector (Agilent Technologies) was used in scan modes to identify the samples. Cholesterol measurements were made in triplicate and normalized to cellular protein content. Cholesterol ester content was calculated by subtracting free cholesterol from total cholesterol measured after saponification. To assess neutral lipid accumulation, cells were fixed in 4% paraformaldehyde and stained with oil red O for 30 min. Staining was recorded on an Olympus X10 microscope equipped with a digital camera.

### Real time RT-PCR analysis

Total RNA was extracted using Trizol B reagent and real-time quantitative RT-PCR (QRT-PCR) was performed using the QuantiTect SYBR Green PCR kit (Qiagen Inc, Valencia, CA) as we previously described [17,18]. Each reaction contained 0.3 μM of CD36, SRA or GAPDH primers, 3 μl of cDNA, SYBR Green, and *HotStarTaq *polymerase. PCR was performed using a BioRad *i*Cycler under the following conditions: 15 min at 95°C, followed by 30 cycles of 30 sec at 95°C, 30 sec at 55°C and 30 sec at 72°C. Each sample was analyzed in triplicate and the amount of CD36, SRA and GAPDH mRNA in each sample was calculated from a standard curve of known template. Data are expressed as the mean number of CD36 and SRA molecules normalized to GAPDH.

### Western analysis

Cells were washed in ice-cold PBS and lysed in radioimmune precipitation buffer containing protease and phosphatase inhibitors. For detection of CD36, 30 μg of protein was run on an 8% denaturing SDS-polyacrylamide gel, transferred to nitrocellulose and blocked overnight in 5% nonfat dry milk and 3% BSA in Tris-buffered saline containing 0.1% Tween 20 (TBS-T) as we previously described [17,26]. Membranes were incubated with a rabbit anti-CD36 antiserum (1:500 dilution) generated in our laboratory [17] for 2 hours, washed three times in TBS-T, and incubated with horseradish peroxidase-conjugated anti-rabbit IgG (1:10,000 dilution) for 1 hour. Blots were washed 3x in TBS-T, exposed to ECL reagent (Amersham Biosciences, Piscataway, NJ), and signal was recorded and quantified using an Alpha Innotech Fluorchem 8800 image analysis system. Blots were stripped and probed with an anti-actin rabbit polyclonal antibody (Santa Cruz Biotechnology) as described above as an internal standard for equivalent loading.

## Results

### β-Amyloid blocks oxidized LDL metabolism and cellular cholesterol accumulation in macrophages and microglia

**Figure 1:**
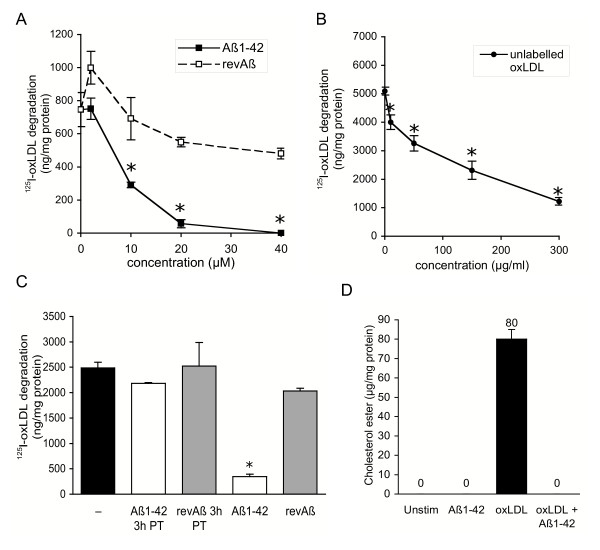
Aβ inhibits lysosomal degradation of oxidized LDL and cholesterol ester accumulation in macrophages. A. Fibrillar Aβ, but not revAβ, dose-dependently inhibits lysosomal degradation of ^125^I-oxLDL by macrophages, similar to unlabeled oxLDL competitor (B). C. Intracellular accumulation of Aβ does not block lysosomal degradation of ^125^I-oxLDL. Macrophages were pretreated with 20 μM Aβ or revAβ for 3 hours to allow intracellular accumulation, washed extensively to remove extracellular peptide and degradation of ^125^I-oxLDL over 5 h was measured in the absence (PT) or presence of additional peptide. D. Aβ blocks cholesterol ester accumulation in oxLDL treated macrophages. Cellular lipids were extracted from macrophages treated with oxLDL (40 μg/ml) for 48 h in the presence or absence of 20 μM Aβ and analyzed by gas-chromatography mass-spectrometry. Cholesterol ester content was normalized to cellular protein. (A-D) Data are the mean of triplicate samples ± standard deviation, *p ≤ 0.005.

Treatment of peritoneal macrophages with Aβ_1-42_, but not *rev*Aβ, dose-dependently inhibited lysosomal degradation of ^125^I-oxLDL (Fig. 1a). Half-maximal inhibition of macrophage ^125^I-oxLDL degradation was achieved with 10 μM Aβ_1-42_. This was equivalent to the inhibitory effect of 15-fold excess of unlabelled oxLDL competitor (Fig. 1b). At 20 μM, Aβ_1-42 _reduced macrophage degradation of ^125^I-oxLDL by up to 90%, while treatment with the same concentration of non-fibrillar *rev*Aβ peptide reduced degradation by only 10%, and this concentration was selected for all further experiments. Because engulfment of Aβ_1-42 _has previously been reported to disrupt endosomal/lysosomal integrity in a neuronal cell line [28], we investigated whether the observed reduction in oxLDL degradation could be attributed to lysosomal accumulation of Aβ_1-42 _which occurs within 1 h of treatment. After exposure to Aβ_1-42 _for 3 hours, macrophages were washed extensively to remove extracellular Aβ_1-42 _and exposed to ^125^I-oxLDL or ^125^I-oxLDL + Aβ_1-42 _for 5 h. While cells continuously exposed to Aβ_1-42 _showed a profound impairment of oxLDL degradation, cells pre-treated with Aβ_1-42 _were similar to untreated and revAβ-treated cells, indicating that intracellular accumulation of Aβ_1-42 _does not block subsequent lysosomal degradation of oxLDL (Fig. 1c).

The inhibition of ^125^I-oxLDL degradation by Aβ_1-42 _would be predicted to reduce cellular cholesterol ester accumulation. Excess unesterified "free" cholesterol is cytotoxic and is thus rapidly converted by the microsomal enzyme acyl-coenzyme A:cholesterol acyltransferase (ACAT) to cholesterol ester for storage. This neutral lipid is retained in cytoplasmic lipid droplets for storage and/or efflux from the cell. Using gas chromatograpy-mass spectrometry, we quantified the cholesterol ester content of macrophages treated with oxLDL in the presence and absence of Aβ_1-42_. As expected, untreated cells did not contain measurable cholesterol ester, while macrophages treated with 40 μg/ml oxLDL for 48 h accumulated approximately 80 μg cholesterol ester/mg cellular protein (Fig. 1d). By contrast, macrophages treated with both oxLDL and Aβ_1-42 _showed no measurable cholesterol ester accumulation after 48 h, similar to untreated cells.

**Figure 2:**
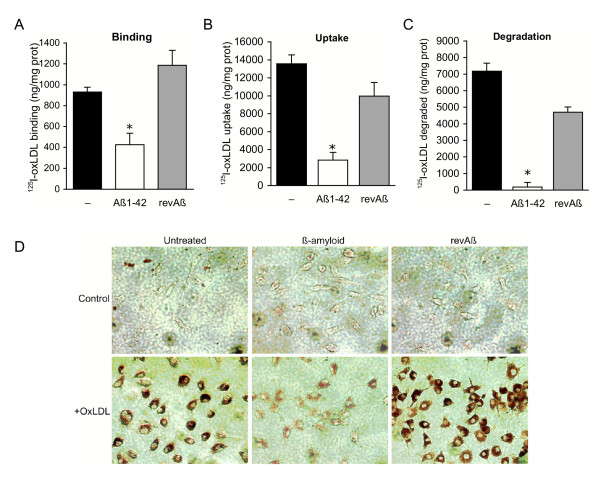
Aβ inhibits oxLDL binding, uptake and degradation in microglia. Treatment of primary microglia with 20 μM fibrillar Aβ, but not revAβ, inhibits ^125^I-oxLDL binding (A), cellular uptake (B) and degradation (C). Data are the mean of triplicate samples ± standard deviation, *p ≤ 0.005. (D) Microglia treated with 20 μM fibrillar Aβ fail to accumulate cholesterol ester in the presence of oxLDL. Microglia were incubated with 40 μg/ml oxLDL for 48 h in the presence and absence of 20 μM Aβ or revAβ peptide and stained with oil red O to visualize neutral lipid. Cells treated with oxLDL alone or in the presence of revAβ demonstrate the accumulation of red-stained lipid droplets in the cytoplasm. By contrast, oil red O staining is greatly reduced in oxLDL and Aβ co-treated microglia. Mag. 200X.

As seen in peripheral macrophages, Aβ_1-42 _substantially inhibited ^125^I-oxLDL binding, uptake, and degradation by primary microglia indicating that it has a similar effect on lipoprotein metabolism in these two myeloid cell types (Fig. 2a,2b,2c). In the presence of 20 μM Aβ_1-42_, microglia demonstrated a 55% reduction in ^125^I-oxLDL binding, an 80% reduction in ^125^I-oxLDL uptake and a 95% reduction of ^125^I-oxLDL degradation. The absence of cholesterol ester in oxLDL treated microglia exposed to Aβ_1-42 _was confirmed by staining cells with the neutral lipid stain oil red O. Microglia treated with oxLDL alone demonstrate oil red O positive lipid droplets in their cytoplasm characteristic of cholesterol ester storage (Fig. 2d). However, in the presence of Aβ_1-42_, oxLDL treated microglia show a dramatic reduction in lipid droplets that is not seen with treatment with the same concentration of *rev*Aβ. As expected, cells treated with Aβ_1-42 _or *rev*Aβ alone do not accumulate cholesterol ester in the absence of exogenously added oxLDL (Fig. 2d). Similar results were observed in macrophages (data not shown). Together, these data demonstrate that Aβ blocks cholesterol ester accumulation in macrophages and microglia by inhibiting oxLDL clearance.

### fAβ downregulates expression of the OxLDL receptor CD36

**Figure 3:**
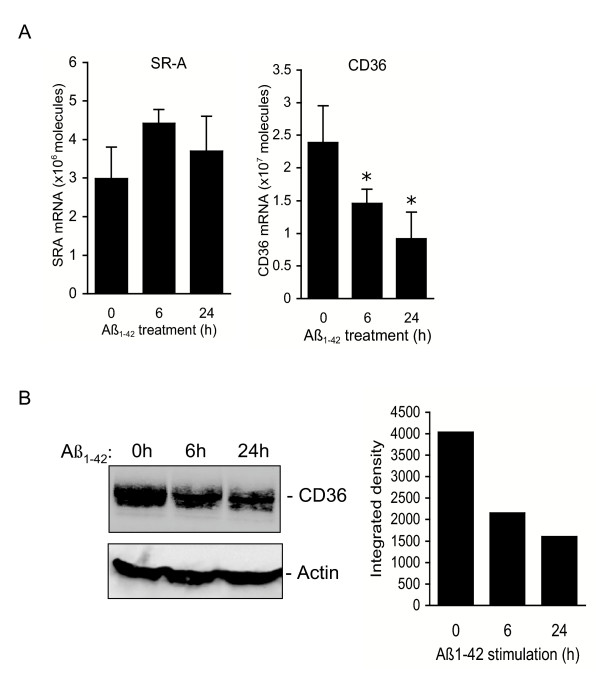
Aβ downregulates expression of the oxLDL receptor CD36. A. Analysis of CD36 and SRA mRNA in peritoneal macrophages treated with Aβ (20 μM) by quantitative RT-PCR. Data represent the mean of triplicate samples ± standard deviation, *p ≤ 0.005. B. Western blot analysis confirming CD36 protein downregulation by Aβ. The signal was recorded and the integrated density value quantified using an Alpha Innotech FluorChem Imager and normalized to actin protein. Data are representative of 2 experiments.

To address the mechanism by which Aβ_1-42 _inhibits oxLDL metabolism, we first evaluated cellular expression of the scavenger receptors SRA and CD36. Fibrillar Aβ_1-42 _reduced expression of CD36 mRNA by 40 and 60% after 6 and 24 h, respectively (Fig. 3a), but showed no effect on macrophage expression of SRA. Western blotting confirmed a 40% decrease in CD36 protein in Aβ_1-42 _treated macrophages (Figure 3b), which would be expected to reduce the ability of these cells to bind oxLDL.

### fAβ competes for oxLDL binding to CD36, but not SRA

**Figure 4:**
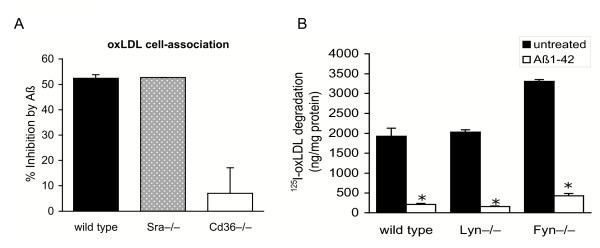
Inhibition of oxLDL cell-association by Aβ requires CD36, but not CD36-associated signal transduction. A. To determine whether SRA or CD36 was essential for Aβ-inhibition of oxLDL metabolism, cell-association of ^125^I-oxLDL was measured in wild type, *Sra*^-/- ^and *Cd36*^-/- ^macrophages in the presence or absence of 20 μM Aβ. While Aβ blocked oxLDL association by approximately 50% in wild type and *Sra*^-/- ^macrophages, this effect was lost in *Cd36*^-/- ^macrophages indicating that CD36 is required for this inhibition. B. Inhibition of ^125^I-oxLDL degradation by Aβ does not utilize the Aβ-CD36 signaling pathway involving Lyn and Fyn kinases. Aβ impaired oxLDL degradation to a similar extent in wild type, and Lyn^-/- ^or Fyn^-/- ^macrophages in which CD36-signaling is impaired, indicating that this signal transduction pathway is not required, Data are the mean of triplicate samples ± standard deviation, *p ≤ 0.005.

β-Amyloid has previously been reported to bind to the class A scavenger receptors SRA I & II and to block uptake of LDL modified by acetylation [14,21]. We employed *Sra *and *Cd36 *single null mice to investigate the role of these receptors in the inhibition of oxLDL clearance by Aβ_1-42_. In addition, we used *Sra*/*Cd36 *double null mice to evaluate the role of SRA/CD36-independent mechanisms, including those of additional scavenger receptor family members. Because of the difficulty of culturing sufficient numbers of primary microglia for binding and degradation experiments, studies involving knock-out mice were performed with peritoneal macrophages. In *Sra*^-/- ^and wild type macrophages Aβ_1-42 _blocked cell association (binding and uptake) of ^125^I-oxLDL by greater than 50%, indicating that this scavenger receptor is not essential for the inhibitory action of Aβ (Fig. 4a). By contrast, in the absence of *Cd36*, impairment of ^125^I-oxLDL cell association by Aβ_1-42 _was reduced to 8%, indicating that this receptor was the primary target of Aβ_1-42 _inhibition (Fig. 4a).

**Figure 5:**
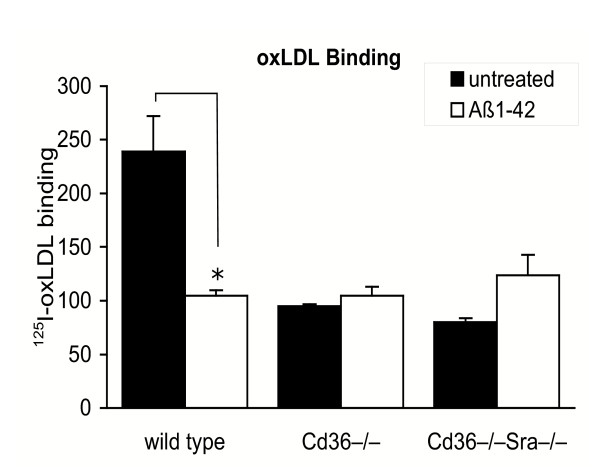
Inhibition of oxLDL binding requires CD36, but not other scavenger receptors. Binding of ^125^I-oxLDL was measured in wild type, *Cd36*^-/- ^or *Cd36/Sra*^-/- ^macrophages in the presence or absence of 20 μM Aβ to assess the role of CD36 and CD36/SRA-independent pathways. In the absence of CD36, oxLDL binding was not reduced by Aβ, indicating that this receptor is the target of Aβ inhibition. Binding of oxLDL via other scavenger receptors, which is measurable in *Cd36/Sra*^-/- ^macrophages, was not inhibited by Aβ. Data are representative of triplicate samples ± standard deviation, *p ≤ 0.005.

The finding that CD36 is required for Aβ_1-42 _inhibition of oxLDL suggests two possible mechanisms of action: (1) direct competition for CD36 binding, or (2) inhibition of oxLDL metabolism as a result of Aβ/CD36 signal transduction. To address whether CD36 signaling inhibits cellular oxLDL degradation, we used macrophages with targeted deletions in two kinases in this pathway, Lyn and Fyn, which have previously been shown to be required for CD36-mediated p44/42 activation, MCP-1 secretion and ROS production [17]. However, as in wild type macrophages, Aβ_1-42 _effectively inhibited ^125^I-oxLDL degradation in *Lyn*^-/- ^and *Fyn*^-/- ^macrophages, suggesting that this signaling pathway does not inhibit oxLDL metabolism (Fig. 4b). Furthermore, treatment of macrophages with the general phosphotyrosine kinase inhibitor genistein did not reverse Aβ_1-42 _inhibition of ^125^I-oxLDL degradation, confirming that phosphotyrosine kinase signaling does not mediate this effect of Aβ_1-42 _(data not shown). Interestingly, in untreated *Fyn*^-/- ^macrophages ^125^I-oxLDL degradation was increased 2-fold (Fig. 4b) indicating that this kinase may play a role in regulating oxLDL uptake. However, despite a doubling of oxLDL degradation in *Fyn*^-/- ^macrophages, this process was still inhibited by Aβ_1-42 _by up to 90%. Together, these experiments suggest that Aβ_1-42 _inhibition of oxLDL metabolism is not the result of CD36-Lyn/Fyn signal transduction and support the hypothesis that Aβ_1-42 _competes for oxLDL binding to CD36. Analysis of ^125^I-oxLDL cell-surface binding showed that Aβ inhibited ^125^I-oxLDL binding by approximately 60% in wild type macrophages (Fig. 5). This inhibitory effect was lost in *Cd36*^-/- ^macrophages, confirming that Aβ inhibited oxLDL binding to this receptor. Of note, wild type macrophages bound 60% more oxLDL than macrophages lacking *Cd36 *as has previously been reported, and this correlated with the percentage reduction of oxLDL binding by Aβ in wild type macrophages (57%), suggesting that the CD36-dependent contribution to oxLDL binding was totally inhibited. To confirm that other myeloid scavenger receptors were not inhibited by Aβ, assesed ^125^I-oxLDL binding in *Sra*^-/-^*Cd36*^-/- ^macrophages. No effect of Aβ was observed in these cells, demonstrating the specificity of Aβ inhibition of oxLDL binding to CD36.

## Discussion

Numerous studies have demonstrated elevated markers of lipid peroxidation in the brains, CSF and plasma of Alzheimer's disease patients, including thiobarbituric acid-reactive substances, 4-hydroxy-2-nonenal (HNE), acrolein and F2-isoprostanes, which are suggestive of a persistent pro-oxidant environment [3,4,9,29,30]. Lipoprotein particles are especially vulnerable to free-radical mediated lipid peroxidation and the resulting peroxy fatty acids are highly unstable, readily decomposing to form peroxy and alkoxy radicals that further promote oxidation. This self-propagating cycle of lipid peroxidation is particularly damaging in lipid-rich tissues such as the brain, and as a result, the innate immune system has evolved mechanisms to rapidly recognize and clear oxidized lipids. The myeloid scavenger receptors are the first lines of defense against these non-native lipids, as well as modified host proteins such as β-amyloid [11,31]. This dual responsibility prompted us to evaluate whether macrophages and microglia would be compromised in their ability to metabolize oxidized lipoproteins in the presence of Aβ. We found that fibrillar Aβ specifically inhibited all phases of oxLDL metabolism, including binding, uptake, degradation and accumulation of cellular cholesterol ester. This was mediated by a selective inhibition of CD36 binding by Aβ, as well as a decrease in CD36 mRNA and protein expression. However, inhibition of oxLDL metabolism was independent of the recently identified Aβ-CD36-signaling cascade, as targeted inactivation of essential downstream kinases did not restore cellular oxLDL degradation. Together, these data demonstrate that oxidized lipoprotein metabolism by CD36 is profoundly impaired in the presence Aβ, and suggest that accumulation of Aβ in the brain and blood vessels in AD would foster the extracellular persistence of these pro-inflammatory lipids, thereby perpetuating lipid peroxidation. Thus, Aβ binding of CD36 in the brain would promote inflammation via two specific mechanisms: (1) through its engagement of signal transduction and microglial recruitment, and (2) through its abrogation of this important clearance pathway for oxidized phospholipid-containing ligands.

In addition to CD36, two other scavenger receptor family members have been shown to be expressed in the brain and to bind Aβ. The Class A scavenger receptors, SRA I and II, and the class B SR-BI are expressed by neonatal microglia, but unlike CD36, these receptors are not expressed by microglia in the normal adult brain [14,15]. However, microglial expression of SRA is increased during AD, and this receptor can mediate both adherence to Aβ and its phagocytosis [14,32,33]. In Sra^-/- ^mice, there is a 60% impairment in microglial binding of Aβ and reactive oxygen production, however, AD-associated brain pathology is not reduced [16,33]. SRA ligands, including acetylated LDL and fucoidan, reduce Aβ uptake by microglia, however these ligands may also affect other receptors [34]. Conversely, Aβ and its soluble precursor protein, sAPPα, inhibit macrophage and microglial uptake of acetylated LDL [14,21,35]. While acetylated LDL is not believed to occur physiologically, other modifications of LDL, such as oxidation, that allow binding to SRA may also be competed by Aβ. However, in our assays Aβ inhibition of oxLDL binding and degradation did not occur via this pathway, similar effects were seen in wild type and Sra^-/- ^cells. By contrast, the effect of Aβ was abolished in the absence of CD36, indicating that this receptor is the target of Aβ action.

The difficulty in isolating human lipoproteins from the CNS has limited their experimental use, however, several groups have shown that oxidized serum lipoproteins, including LDL, HDL and VLDL, are toxic to neurons [36-39], and both oxLDL and oxidized CSF lipoproteins disrupt neuronal microtubule organization, a pathogy characteristic of the AD brain [6,38,40]. Thus, the loss of CD36-mediated oxidized lipoprotein clearance in the presence of Aβ_1-42 _would be predicted to foster inflammation and tissue injury. While we have shown that Aβ blocks CD36 binding of oxLDL, and its subsequent degradation, we would predict that similar results would be found with oxidized lipoproteins isolated from the CNS, astrocytes or microglia. Although serum and brain lipoprotein particles differ in their apolipoprotein composition [23,41-44], they contain similar amounts of cholesterol, cholesterol ester and phospholipid. CD36 has been shown to recognize a phospholipid moiety of oxidized lipoproteins, primarily oxidized phosphatidylcholine, which is abundant in CSF lipoproteins [22,41]. The presence of a pro-oxidant environment in AD would be expected to generate similar modifications of CSF lipoproteins and lipoproteins isolated from AD-affected individuals have, in fact, been shown to be more susceptible to oxidation [5,6]. Inhibition of the primary clearance pathway for oxidized lipoproteins would be predicted to promote inflammation and persistence of lipid peroxidation.

Disruption of oxidized lipoprotein metabolism by Aβ may also be relevant in the context of atherosclerosis. Cholesterol oxidation products generated during the inflammatory component of atherosclerosis have been shown to accelerate β-amyloid fibril formation [10,45]. Furthermore, a recent study identified Aβ advanced human atherosclerotic plaques [46]. Our data suggests that the presence of Aβ in the artery wall may both prevent macrophage oxidized LDL uptake via CD36, thereby promoting β-amyloid fibril formation and activating CD36-signaling [47]. It has recently been shown that Aβ-CD36-signaling leads to the expression of cytokines and chemokines, including IL-1β, TNFα, MCP-1, MIP-1α and β and MIP-2 [17-19]. Activation of this signaling cascade would be predicted to promote inflammation, as well as atherosclerotic plaque progression. Indeed, overexpression of a mutant human amyloid β-precursor protein in an atherosclerosis-susceptible mouse strain (B6Tg2576) led to significantly increased levels of atherosclerosis, which correlated positively with cerebral Aβ deposits [48]. Of particular interest, when these mice were maintained on a normal chow diet that did not induce atherosclerosis in wild type littermates, B6Tg2576 mice developed early atherosclerotic lesions in the aortic root, suggesting that Aβ promotes atherogenesis. The convergence of risk factors for AD and atherosclerosis suggest that these chronic inflammatory diseases may have overlapping mechanisms of pathogenesis in which cholesterol levels and lipid peroxidation play a central role.

## List of abbreviations used

Aβ, β-amyloid peptide 1–42; ACAT, acyl-coenzyme A:cholesterol acyltransferase; AD, Alzheimer's disease; CSF, cerebral spinal fluid; DMEM, Dubelcco's modified Eagle medium; FCS, fetal calf serum; fAβ, fibrillar Aβ; GC-MS, gas chromatography-mass spectrometry HNE, 4-hydroxy-2-nonenal; oxLDL, oxidized low density lipoprotein; *rev*Aβ, reverse β-amyloid peptide 42-1; SRA, scavenger receptor A; SR-BI, scavenger receptor B I.

## Competing interests

The authors declare that they have no competing interests.

## Authors' contributions

VVK performed the measurements of ^125^I-oxLDL binding, uptake and degradation, and participated in the design of the study and analysis of results. LAM and TK isolated the primary microglia and macrophages, performed western blots, quantitative RT-PCR, and measurements of 125I-oxLDL binding, uptake and degradation. AAT performed measurements of ^125^I-oxLDL binding, uptake and degradation. KJM conceived of the study, participated in its design and wrote the manuscript. All authors read and approved the final manuscript.

## References

[B1] Marx J (2001). Alzheimer's disease. Bad for the heart, bad for the mind?. Science.

[B2] Ross R (1999). Atherosclerosis--an inflammatory disease. N Engl J Med.

[B3] Dei R, Takeda A, Niwa H, Li M, Nakagomi Y, Watanabe M, Inagaki T, Washimi Y, Yasuda Y, Horie K, Miyata T, Sobue G (2002). Lipid peroxidation and advanced glycation end products in the brain in normal aging and in Alzheimer's disease. Acta Neuropathol (Berl).

[B4] Butterfield DA, Lauderback CM (2002). Lipid peroxidation and protein oxidation in Alzheimer's disease brain: potential causes and consequences involving amyloid beta-peptide-associated free radical oxidative stress. Free Radic Biol Med.

[B5] Schippling S, Kontush A, Arlt S, Buhmann C, Sturenburg HJ, Mann U, Muller-Thomsen T, Beisiegel U (2000). Increased lipoprotein oxidation in Alzheimer's disease. Free Radic Biol Med.

[B6] Bassett CN, Neely MD, Sidell KR, Markesbery WR, Swift LL, Montine TJ (1999). Cerebrospinal fluid lipoproteins are more vulnerable to oxidation in Alzheimer's disease and are neurotoxic when oxidized ex vivo. Lipids.

[B7] Jarvik GP, Wijsman EM, Kukull WA, Schellenberg GD, Yu C, Larson EB (1995). Interactions of apolipoprotein E genotype, total cholesterol level, age, and sex in prediction of Alzheimer's disease: a case-control study. Neurology.

[B8] Kuo YM, Emmerling MR, Bisgaier CL, Essenburg AD, Lampert HC, Drumm D, Roher AE (1998). Elevated low-density lipoprotein in Alzheimer's disease correlates with bain abeta 1-42 levels. Biochem Biophys Res Commun.

[B9] Bassett CN, Montine TJ (2003). Lipoproteins and lipid peroxidation in Alzheimer's disease. J Nutr Health Aging.

[B10] Zhang Q, Powers ET, Nieva J, Huff ME, Dendle MA, Bieschke J, Glabe CG, Eschenmoser A, Wentworth PJ, Lerner RA, Kelly JW (2004). Metabolite-initiated protein misfolding may trigger Alzheimer's disease. Proc Natl Acad Sci U S A.

[B11] Peiser L, Mukhopadhyay S, Gordon S (2002). Scavenger receptors in innate immunity. Curr Opin Immunol.

[B12] Kunjathoor VV, Febbraio M, Podrez EA, Moore KJ, Andersson L, Koehn S, Rhee JS, Silverstein R, Hoff HF, Freeman MW (2002). Scavenger receptors class A-I/II and CD36 are the principal receptors responsible for the uptake of modified low density lipoprotein leading to lipid loading in macrophages. J Biol Chem.

[B13] Coraci IS, Husemann J, Berman JW, Hulette C, Dufour JH, Campanella GK, Luster AD, Silverstein SC, El Khoury J (2002). CD36, a class B scavenger receptor, is expressed on microglia in Alzheimer's disease brains and can mediate production of reactive oxygen species in response to b-amyloid fibrils. Am J Pathol.

[B14] El Khoury J, Hickman SE, Thomas CA, Cao L, Silverstein SC, Loike JD (1996). Scavenger receptor-mediated adhesion of microglia to beta-amyloid fibrils. Nature.

[B15] Husemann J, Loike JD, Kodama T, Silverstein SC (2001). Scavenger receptor class B type I (SR-BI) mediates adhesion of neonatal murine microglia to fibrillar beta-amyloid. J Neuroimmunol.

[B16] Huang F, Buttini M, Wyss-Coray T, McConlogue L, Kodama T, Pitas RE, Mucke L (1999). Elimination of the class A scavenger receptor does not affect amyloid plaque formation or neurodegeneration in transgenic mice expressing human amyloid protein precursors. Am J Pathol.

[B17] Moore KJ, El Khoury J, Medeiros LA, Terada K, Geula C, Luster AD, Freeman MW (2002). A CD36-initiated signaling cascade mediates inflammatory effects of beta-amyloid. J Biol Chem.

[B18] El Khoury JB, Moore KJ, Means TK, Leung J, Terada K, Toft M, Freeman MW, Luster AD (2003). CD36 mediates the innate host response to beta-amyloid. J Exp Med.

[B19] Bamberger ME, Harris ME, McDonald DR, Husemann J, Landreth GE (2003). A cell surface receptor complex for fibrillar beta-amyloid mediates microglial activation. J Neurosci.

[B20] Ricciarelli R, D'Abramo C, Zingg JM, Giliberto L, Markesbery W, Azzi A, Marinari UM, Pronzato MA, Tabaton M (2004). CD36 overexpression in human brain correlates with beta-amyloid deposition but not with Alzheimer's disease. Free Radic Biol Med.

[B21] Paresce DM, Ghosh RN, Maxfield FR (1996). Microglial cells internalize aggregates of the Alzheimer's disease amyloid beta-protein via a scavenger receptor. Neuron.

[B22] Podrez EA, Hoppe G, O'Neil J, Hoff HF (2003). Phospholipids in oxidized LDL not adducted to apoB are recognized by the CD36 scavenger receptor. Free Radic Biol Med.

[B23] Cole GM, Beech W, Frautschy SA, Sigel J, Glasgow C, Ard MD (1999). Lipoprotein effects on Abeta accumulation and degradation by microglia in vitro. J Neurosci Res.

[B24] Suzuki H, Kurihara Y, Takeya M, Kamada N, Kataoka M, Jishage K, Ueda O, Sakaguchi H, Higashi T, Suzuki T, Takashima Y, Kawabe Y, Cynshi O, Wada Y, Honda M, Kurihara H, Aburatani H, Doi T, Matsumoto A, Azuma S, Noda T, Toyoda Y, Itakura H, Yazaki Y, Kodama T (1997). A role for macrophage scavenger receptors in atherosclerosis and susceptibility to infection. Nature.

[B25] Moore KJ, Andersson LP, Ingalls RR, Monks BG, Li R, Arnaout MA, Golenbock DT, Freeman MW (2000). Divergent response to LPS and bacteria in CD14-deficient murine macrophages. J Immunol.

[B26] Moore KJ, Rosen ED, Fitzgerald ML, Randow F, Andersson LP, Altshuler D, Milstone DS, Mortensen RM, Spiegelman BM, Freeman MW (2001). The role of PPAR-gamma in macrophage differentiation and cholesterol uptake. Nat Med.

[B27] Brown MS, Basu SK, Falck JR, Ho YK, Goldstein JL (1980). The scavenger cell pathway for lipoprotein degradation: specificity of the binding site that mediates the uptake of negatively-charged LDL by macrophages. J Supramol Struct.

[B28] Yang AJ, Chandswangbhuvana D, Margol L, Glabe CG (1998). Loss of endosomal/lysosomal membrane impermeability is an early event in amyloid Abeta1-42 pathogenesis. J Neurosci Res.

[B29] Arlt S, Beisiegel U, Kontush A (2002). Lipid peroxidation in neurodegeneration: new insights into Alzheimer's disease. Curr Opin Lipidol.

[B30] Montine TJ, Neely MD, Quinn JF, Beal MF, Markesbery WR, Roberts LJ, Morrow JD (2002). Lipid peroxidation in aging brain and Alzheimer's disease. Free Radic Biol Med.

[B31] Husemann J, Loike JD, Anankov R, Febbraio M, Silverstein SC (2002). Scavenger receptors in neurobiology and neuropathology: their role on microglia and other cells of the nervous system. Glia.

[B32] Christie RH, Freeman M, Hyman BT (1996). Expression of the macrophage scavenger receptor, a multifunctional lipoprotein receptor, in microglia associated with senile plaques in Alzheimer's disease. Am J Pathol.

[B33] Chung H, Brazil MI, Irizarry MC, Hyman BT, Maxfield FR (2001). Uptake of fibrillar beta-amyloid by microglia isolated from MSR-A (type I and type II) knockout mice. Neuroreport.

[B34] Kim WS, Ordija CM, Freeman MW (2003). Activation of signaling pathways by putative scavenger receptor class A (SR-A) ligands requires CD14 but not SR-A. Biochem Biophys Res Commun.

[B35] Santiago-Garcia J, Mas-Oliva J, Innerarity TL, Pitas RE (2001). Secreted forms of the amyloid-beta precursor protein are ligands for the class A scavenger receptor. J Biol Chem.

[B36] Keller JN, Hanni KB, Kindy MS (2000). Oxidized high-density lipoprotein induces neuron death. Exp Neurol.

[B37] Keller JN, Hanni KB, Gabbita SP, Friebe V, Mattson MP, Kindy MS (1999). Oxidized lipoproteins increase reactive oxygen species formation in microglia and astrocyte cell lines. Brain Res.

[B38] Keller JN, Hanni KB, Markesbery WR (1999). Oxidized low-density lipoprotein induces neuronal death: implications for calcium, reactive oxygen species, and caspases. J Neurochem.

[B39] Paradis E, Clement S, Julien P, Ven Murthy MR (2003). Lipoprotein lipase affects the survival and differentiation of neural cells exposed to very low density lipoprotein. J Biol Chem.

[B40] Neely MD, Swift LL, Montine TJ (2000). Human, but not bovine, oxidized cerebral spinal fluid lipoproteins disrupt neuronal microtubules. Lipids.

[B41] Pitas RE, Boyles JK, Lee SH, Hui D, Weisgraber KH (1987). Lipoproteins and their receptors in the central nervous system. Characterization of the lipoproteins in cerebrospinal fluid and identification of apolipoprotein B,E(LDL) receptors in the brain. J Biol Chem.

[B42] Xu Q, Li Y, Cyras C, Sanan DA, Cordell B (2000). Isolation and characterization of apolipoproteins from murine microglia. Identification of a low density lipoprotein-like apolipoprotein J-rich but E-poor spherical particle. J Biol Chem.

[B43] LaDu MJ, Gilligan SM, Lukens JR, Cabana VG, Reardon CA, Van Eldik LJ, Holtzman DM (1998). Nascent astrocyte particles differ from lipoproteins in CSF. J Neurochem.

[B44] Koch S, Donarski N, Goetze K, Kreckel M, Stuerenburg HJ, Buhmann C, Beisiegel U (2001). Characterization of four lipoprotein classes in human cerebrospinal fluid. J Lipid Res.

[B45] Stanyer L, Betteridge DJ, Smith CC (2004). Potentiation of beta-amyloid polymerisation by low-density lipoprotein enhances the peptide's vasoactivity. Biochim Biophys Acta.

[B46] De Meyer GR, De Cleen DM, Cooper S, Knaapen MW, Jans DM, Martinet W, Herman AG, Bult H, Kockx MM (2002). Platelet phagocytosis and processing of beta-amyloid precursor protein as a mechanism of macrophage activation in atherosclerosis. Circ Res.

[B47] Moore KJ, El Khoury J, Medeiros LA, Terada K, Geula C, Luster AD, Freeman MW (2002). A CD36-initiated signaling cascade mediates inflammatory effects of beta -amyloid. J Biol Chem.

[B48] Li L, Cao D, Garber DW, Kim H, Fukuchi K (2003). Association of aortic atherosclerosis with cerebral beta-amyloidosis and learning deficits in a mouse model of Alzheimer's disease. Am J Pathol.

